# Large-Area Fabrication of LIPSS for Wetting Control Using Multi-Parallel Femtosecond Laser Processing

**DOI:** 10.3390/ma15165534

**Published:** 2022-08-11

**Authors:** Simonas Indrišiūnas, Evaldas Svirplys, Mindaugas Gedvilas

**Affiliations:** Department of Laser Technologies (LTS), Center for Physical Sciences and Technology (FTMC), Savanorių Ave. 231, LT-02300 Vilnius, Lithuania

**Keywords:** multi-parallel laser beam processing, LIPSS, wettability, superhydrophobicity, EN 1.4301 stainless steel

## Abstract

In this research, the wetting property control of a stainless-steel surface, structured using parallel processing via an array of 64-femtosecond laser beams, is presented. The scanning of an 8 × 8-beam array over the sample was used to uniformly cover the large areas with LIPSS. The static water contact angle and the LIPSS period dependence on processing parameters were investigated. The wettability control of water droplets on laser-patterned stainless steel, ranging from contact angles of ~63°, similar to those of the plain surface, to the superhydrophobic surface with contact angles > 150°, was achieved. The relationship between the static water contact angle and the LIPSS parameters in the Fourier plane was investigated.

## 1. Introduction

Self-organized laser-fabricated semi-periodical surface structures such as laser-induced periodic surface structures (LIPSS) or various micro-cone or pillar patterns are widely investigated as possible means of producing a surface with the needed wettability [1] or tribological [2] properties. They are utilized both as self-sufficient structures for surface-property control or as elements of a hierarchical structure with larger elements made, e.g., via laser ablation of the regular pattern [3,4]. It is accepted that at least large-period LIPSS are formed by the interaction of the incident laser beam with an electromagnetic wave scattered at the rough surface and may involve the excitation of surface plasmon polaritons. However, not all LIPSS-related phenomena are fully explained, especially the formation of LIPSS structures with periods smaller than half the irradiation wavelength. The formation of LIPSS requires ultrashort laser pulses; thus, femtosecond and sub-picosecond pulses are most widely used for their generation [5].

To fabricate a self-organized texture, an area is scanned with a laser beam using high pulse overlap, i.e., in [6], a femtosecond laser beam scanned with a large overlap was used to produce self-organized conical protrusions covered with LIPSS on the austenitic AISI 316L steel surface. After the silanization of the samples, they demonstrated both high static water contact angle (SWCA) values (>150°) and low roll-off angles (<10°). Although the fabrication of LIPSS requires relatively low laser fluence (close to the ablation threshold fluence) to produce a high-regularity structure, a small laser-spot size (comparable to the mean free path of surface plasmon polaritons in the material) still has to be used to avoid a loss in cohesion over the laser spot [7]. In such a case, parallel laser processing via multi-beam array can be used for large-area fabrication, using more than one laser beam. In most of the multi-beam setups, a diffractive optical element (DOE) is used to split one laser beam into many [8,9]. DOEs can withstand high average laser power or pulse energy, and the technology of their fabrication is well-developed. For the fast partial coverage of a large area with LIPSS, the direct laser interference patterning (DLIP) technique can be used [10]. In [11], the wettability properties of hierarchical microstructures, produced using DLIP with picosecond pulses, were investigated. The hierarchical pillar structure was obtained by using a 5.5 µm period 2-beam DLIP line pattern to scan the sample two times at perpendicular angles. Between the micropillars, low- (200 nm) and high- (800 nm)-spatial-period LIPSS were formed, parallel and perpendicular to the laser beam polarization direction, respectively. It was determined that after 50 days, the hierarchical microstructure had superhydrophobic properties (a static water contact angle higher than 150°; contact angle hysteresis higher than 30°). Additionally, in [11], the importance of the dual-scale structure was demonstrated: some samples were treated using a pulsed nanosecond laser to remove LIPSS via melting (resulting in a single-scale structure). After such treatment, the static water contact angle was reduced to 110°, and the contact angle hysteresis increased to 45°. Additionally, a DOE with a galvo scanner can be used to obtain both high-speed beam control and parallel capability [8]. In [12], a very dense 51 × 51 laser-spot array was used with a galvo scanner to produce closely packed spots containing LIPSS, similar to those in the DLIP patterning.

In this work, the scanning of an 8 × 8-beam array over the sample was used to uniformly cover the large areas of the stainless-steel surface with LIPSS. The properties of LIPSS and the relationships between them and the static water contact angle were investigated, and the relationships between the static water contact angle and the first- and second-order maxima in the Fourier space, corresponding to the various period LIPSS, were determined.

## 2. Methods and Materials

### 2.1. Laser Patterning Setup

**Figure 1:**
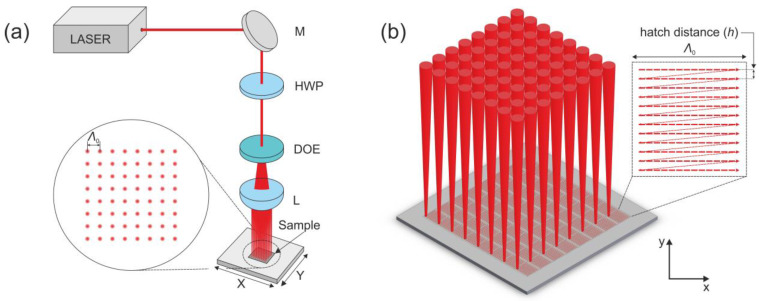
(**a**) Optical setup for the generation of the 8 × 8-beam array. *Λ*_0_—period of the beam array, M—mirror, HWP—half-wave plate, DOE—diffractive optical element, L—focusing lens. X and Y denote the linear positioning stages. (**b**) Illustration of area scanning using 8 × 8-beam array. Inset is an illustration of unidirectional sequential beam-hatching pattern simultaneously performed for each of 64 beams.

The area-scanning experiment was performed using a high-repetition-rate femtosecond laser, Pharos, from Light Conversion (60–600 kHz, 515 nm, max. pulse energy 60 µJ at 100 kHz) with a pulse duration of 300 fs. The optical setup is displayed in Figure 1.

A 5 mm-diameter (at 1/*e*^2^ level) laser beam was directed toward the diffractive optical element (MS-384-515-Y-A; HOLO/OR; full angle 1.92° × 1.92°; separation angle 0.27°; design wavelength 515 nm), which divided the laser beam into 64 (8 × 8) beams. The beams were focused on the sample using the 25 mm focal length aspheric lens with a numerical aperture (NA) of 0.5. The spot size radius *w*_0_, measured using the Liu method [13], was 3.5 µm. The peak laser irradiation fluence *F*_0_ was calculated using the expression *F*_0_ = 2*E*_p_/(π*w*_0_^2^), where *E*p is the laser-pulse energy, related to the average power *P* and pulse repetition rate *f: E*p = *P*/*f.* The average laser power was measured with Ophir F150(200)A-CM-16 sensor.

The laser beam position in relation to the sample was changed by moving the sample in directions perpendicular to the beams in a beam array using linear translation axes (Aerotech ANT 130-160 XY). The distance from the sample to the focal position was also controlled using a motorized positioning stage. Using the described patterning setup, the distance between the laser spots on the sample surface (beam array period) was *Λ*_0_ = 118 µm. The beam array was used to fill square areas with scan lines, as shown in Figure 1b. The laser beam array was used for the uniform structuring of certain areas (hatching) via beam array scanning in an area smaller than or equal to *Λ*_0_. The size of a single scan area was slightly lower than *Λ*_0_ (by 4 µm) to reduce overlap between the areas scanned by each beam. So, in one fabrication step, an approximately 1 mm^2^ area was patterned by moving the stages in a 64-times-smaller area than would be needed when using a single laser beam. The patterned area was further increased by putting the patterned areas next to each other. The laser beam was linearly polarized, and the direction of the polarization vector was parallel to the beam scanning direction. Beam scanning was performed in the x (horizontal) direction, and the hatching was performed in the y (vertical) direction, as shown in Figure 1b. In the experiment, the peak laser irradiation fluence *F*_0_ and the distance between the scan lines, *h* (shown in Figure 1b), were varied. Inside the scan lines, a constant pulse density of 10,000 pulses per millimeter was kept for all samples. For all samples, a constant speed of 1 mm/s was used. Using fixed *F*_0_ and *h* values 3 × 3 mm^2^ sized areas were patterned. In a total 121 area, utilizing 12 hatch values and 11 fluence values, was produced as the final sample.

A stream of compressed air parallel to the sample surface was used to prevent ablation products from polluting the focusing lens. However, its influence on the sample temperature was negligible.

### 2.2. Measurement of the Static Water Contact Angle

Before the measurement of the static water contact angle (SWCA), laser-patterned samples were cleaned in acetone in an ultrasonic bath for 5 min. The SWCA was measured by placing the a 4 µL-volume water drop on top of the patterned area using a micropipette (Transferpette^®^ S, volume 0.5–10 µL, Brand GmbH). The sample with a deposited water drop was illuminated from one side using a high-parallelism-diode backlight, and, from the opposite side, an image of the drop was taken using a digital camera. The SWCA value was evaluated from the image using the LBADSA [14] plug-in of ImageJ software [15]. Samples on which the water drop could not be placed using a micropipette due to the high repulsion forces between the drop and the sample surface were considered superhydrophobic (ultrahigh SWCA). The SWCAs of the samples were measured approximately 60 days after laser texturing to allow settling down of the surface chemical composition.

### 2.3. Characterization of the LIPSS Period and Depth

The period of the LIPSS structures (ripples) was evaluated by transforming a scanning electron microscope (SEM) image using two-dimensional fast Fourier transform (2D-FFT) and measuring the distances between the non-central peaks in the Fourier plane. The 2D-FFT procedure was performed using the open-source software Gwyddion [16]. The peaks were fitted with Lorentz functions before the distances were measured. The LIPSS period *Λ*_LIPSS_ was calculated from the expression *Λ*_LIPSS_ = 2/(*f*_2_ − *f*_1_), where *f*_1_ and *f*_2_ were the positions of the two peaks, which were at the same distance from the central peak on both sides. If more than one pair of peaks were observed, the procedure was repeated for each peak pair.

The depth of the LIPSS structures was measured using an atomic force microscope (AFM). For this task, a Dimension Edge AFM from Bruker in tapping mode was utilized For measurements of nano-scale laser-induced ripples. A commercial silicon probe with a tip diameter of <10 nm (force constant of −40 Nm^−1^) was used.

### 2.4. Sample Material

Polished 0.8 mm-thick steel plates (1.4301, polished super mirror No. 8) were used for the laser patterning. The initial roughness of the steel surface was *S*_a_ = 49 nm. Before the laser processing, the plate surface was wiped with a tissue moistened with acetone.

## 3. Results and Discussion

**Figure 2:**
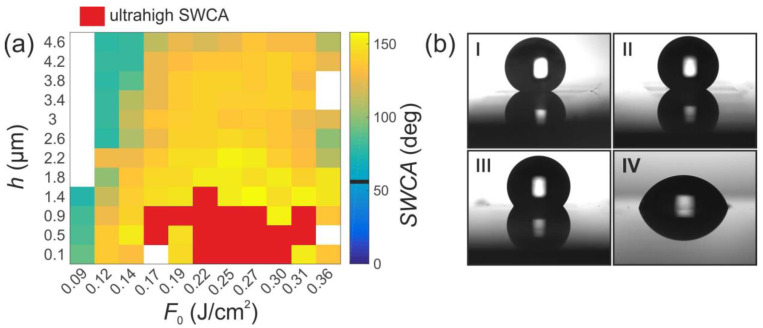
(**a**) Dependence of the static water contact angle (SWCA) of the samples prepared using beam array area hatching on the laser fluence F_0_ and hatch distance h; black line on the color bar indicates SWCA value of an untextured surface (63.3 ± 1.9°). (**b**) Water drops on the surfaces, structured using various parameters: (**I**)—h = 1.4 µm, F_0_ = 0.27 J/cm^2^; (**II**)—h = 1.8 µm, F_0_ = 0.25 J/cm^2^; (**III**)—h = 4.2 µm, F_0_ = 0.25 J/cm^2^; and (**IV**)—h = 1.4 µm, F_0_ = 0.09 J/cm^2^.

In Figure 2, the measured dependence of the SWCA on the fluence *F*_0_ and the hatch distance *h* parameters is provided. The SWCA values varied from modestly higher than the SWCA of the nontextured surface to values of 150°, and ultra-high static contact angles when it was not possible to place the water droplet on the surface using a micropipette. The SWCA of an untextured steel surface was 63.3 ± 1.9°. This value is indicated in the color bar in Figure 2a by a thick black line. In the investigated range of parameters, moderate fluencies and low hatch distance were most favorable for obtaining a structure with an ultrahigh static contact angle. The low SWCA values at low fluencies (0.09; 0.12 J/cm^2^) and the large hatch distances can be attributed to the Wenzel wetting state [17], in which water fills the LIPSS grooves, and the SWCA values are similar to those of the untreated surface. The increase in the SWCA values at higher fluencies and the small hatch distances correspond to the Cassie–Baxter wetting state [18], in which the water rests on the top of the ripple ridges with air trapped beneath.

**Figure 3:**
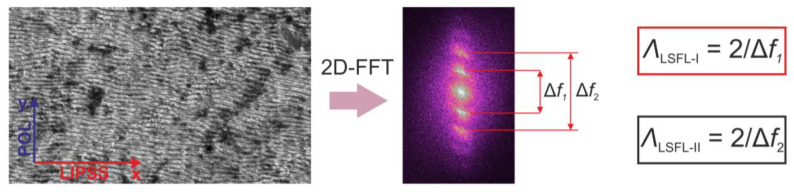
Graphical illustration of evaluation procedure of the LIPSS period. Arrows in the leftmost image show the directions of laser beam polarization (POL) and LIPSS grooves (LIPSS).

The relationship between the surface structure and the SWCA was investigated by evaluating the LIPSS properties from the 2D-FFT transform of the LIPSS SEM images (Figure 3). For most of the samples, two pairs of peaks on both sides of the central peak could be observed in the Fourier plane. This indicates that LIPSS with two distinctive periods were produced. The periods were approximately 0.2 µm and 0.35 µm (0.39*λ* and 0.68*λ*, respectively) and slightly varied with the laser-processing parameters. Usually, LIPSS are classified as high-spatial-frequency LIPSS (HSFL) when their spatial period is significantly smaller than the laser wavelength *λ* (HSFL < *λ*/2), and as low-spatial-frequency LIPSS (LSFL) when their spatial period is close to *λ* [19]. However, certain irradiation conditions may lead to the splitting of the LSFL ripples, and LSFL is transformed into an LIPSS structure with a halved spatial period. LSFL ripples with a spatial period close to the irradiation wavelength *λ* may be denoted as LSFL-I, and LSFL ripples with a period of ~*λ*/n, where n is a positive nonzero integer, may be denoted as LSFL-II [5,20].

**Figure 4:**
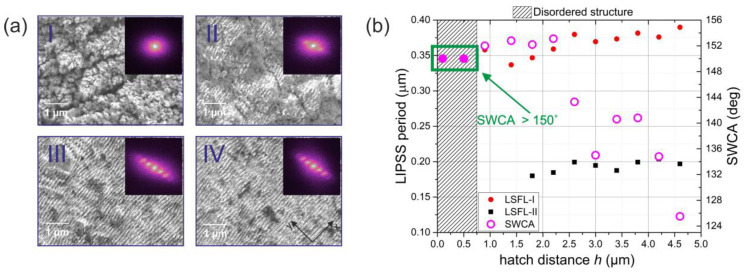
(**a**) SEM micrographs of the steel surfaces, laser patterned using various h values: (**I**)—0.1 µm, (**II**)—0.9 µm, (**III**)—3.4 µm, and (**IV**)—4.6 µm (each SEM micrograph inset shows its 2D-FFT image). All samples were prepared using constant fluence (0.25 J/cm^2^). (**b**) Dependence of the LIPSS period and SWCA value on the hatch distance h.

As can be seen from the 2D-FFT images in Figure 4a, both kinds of ripple were oriented in the same direction (the peaks in the 2D-FFT image can be connected through the central peak using a single line). This direction was perpendicular to the polarization. For most of the metals, it was reported in the literature that the LSFL ripples were perpendicular to the laser polarization direction, and the HSFL ripples parallel [1,19]. So, it may be concluded that both of the obtained ripples with distinct periods were LSFL ripples and split LSFL ripples, and they will be referred to as LSFL-I (larger period; 0.35 µm) and LSFL-II (smaller period; 0.2 µm) in the remaining text.

In Figure 4b, the dependencies of the LSFL-I and LSFL-II LIPSS periods and the SWCA on the hatch distance *h* are provided. The SWCA value increases with decreasing hatch distance, and ultra-high contact angle values, at which it was not possible to stick the water drop to the sample surface using a micropipette, were reached when *h* < 0.75 µm. The relation between small hatch distance and high SWCA can be explained by the continuous increase in the structure surface waviness when decreasing *h*. As shown in the SEM micrographs in Figure 4a, at high *h* values, a relatively smooth structure, mostly consisting of regular LIPSS, is obtained (Figure 4a-III,IV), whereas with decreasing hatch distance, larger laser-induced structures—microcones—began to appear (Figure 4a-II). The disappearance of one pair of peaks, situated further from the central peak, in the 2D-FFT images obtained from the SEM micrographs shows that only LSFL-I ripples are formed in this case. In the regime where ultrahigh SWCAs are obtained, the structure consists of several micrometer-sized increases covered with fine additional structures (Figure 4a-I). In the 2D-FFT image of this structure, the peaks corresponding to the regular LIPSS structure entirely disappear. The ultrahigh SWCA values obtained in this case may be explained by the hierarchical nature of such a disordered structure.

The period of the LIPSS increased slightly with increasing hatch distance (Figure 4a). This result agrees with the literature, i.e., in [21], it is shown that the LIPSS period grows with increasing laser intensity factor, which is directly proportional to laser fluence and inversely proportional to pulse pitch (scanning speed divided by pulse repetition rate). However, no clear relation between the LIPSS period and the SWCA was found.

**Figure 5:**
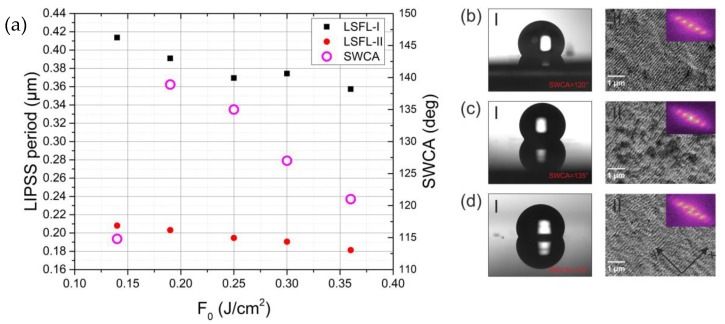
(**a**) Dependence of the LIPSS period and SWCA of the samples prepared using beam array area hatching, on the laser fluence F_0_. (**b**–**d**) Images of the water droplets (**I**) and SEM micrographs (**II**) on the surfaces textured using F_0_ = 0.12 J/cm^2^, 0.25 J/cm^2^, and 0.36 J/cm^2^, respectively (h = 1.8 µm was equal for all samples); insets in the SEM micrographs show their 2D-FFT images.

In Figure 5a, the dependence of the LIPSS period and the SWCA on the laser fluence *F*_0_ (for constant hatch distance *h* = 1.8 µm) is provided. The SWCA dependence on the *F*_0_ has a peak–optimal fluence for high SWCA. The existence of the optimal fluence, needed to obtain high SWCAs, can be explained by investigating the small-scale structure. As shown in the SEM micrographs of the areas, processed using constant hatch distance (Figure 5b–dII), the low (Figure 5b-II) and high (Figure 5d-II) fluence provide quite a regular LIPSS structure. However, at middle fluencies, the structure also has larger-scale roughness (Figure 5c-II), which could be responsible for the larger SWCA. Additionally, it has to be noted that at a low fluence, gaps between the areas, patterned with each beam of the array, appeared due to the fact that only the tip of the Gaussian beam had enough energy to modify the material. This fluence region corresponds to the low-SWCA zone on the left side of Figure 5a.

**Figure 6:**
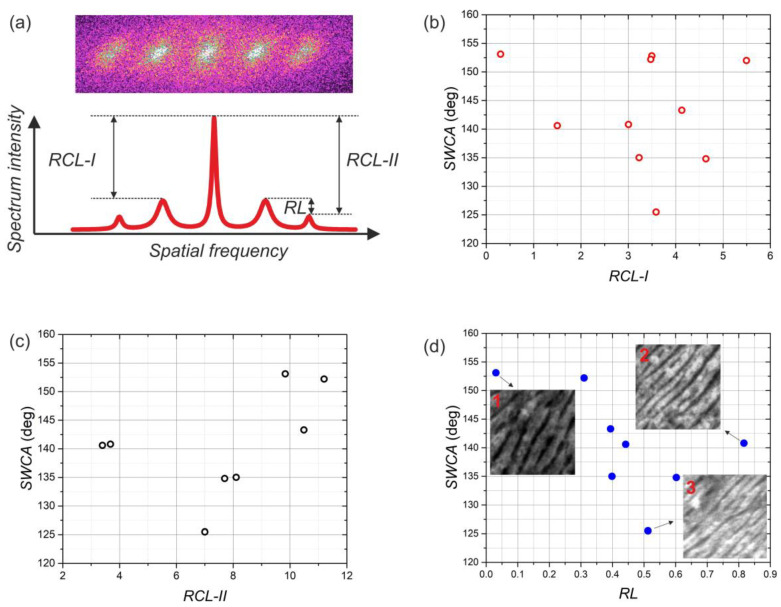
(**a**) Evaluation of the ratios between LSFL-I peak height and central peak height (RCL-I), between LSFL-II peak height and central peak height (RCL-II), and between the LSFL-I and LSFL-II (RL) in the Fourier space. (**b**) Dependence of SWCA on the ratio between LSFL-I peaks and central peak in the Fourier space (RCL-I). (**c**) Dependence of SWCA on the ratio between LSFL-II peaks and central peak in the Fourier space (RCL-II). (**d**) Dependence of SWCA on the ratio between LSFL-I and LSFL-II peaks (RL). Samples were fabricated using the same fluence (0.041 J/cm^2^) and variable hatch distance.

The relationships between the LIPSS properties, laser-processing parameters and the static water contact angle were further investigated by measuring various peak ratios in the 2D-FFT spectra. The ratio between the LSFL-I peaks and the central peak was denoted as RCL-I, the ratio between the LSFL-II peaks and the central peak as RCL-II, and the ratio between the LSFL-II and LSFL-I peaks was denoted as RL (Figure 6a). The dependence of the SWCA on the RCL-I and RCL-II ratios for the samples, fabricated using variable hatch distance (from 0.9 to 4.6 μm) and 0.25 J/cm^2^ fluence, are presented in Figure 6b,c, respectively. There is no clear relation between the SCWA and RCL-I. However, low SCWA values correlate with RCL-II ratio values close to 7. Additionally, the dependence of the SWCA on the ratio between the LSFL-II and LSFL-I peak heights (RL) depicted in Figure 6d shows that when this ratio is about 0.5, the SWCA values are the lowest. So, it appears that, in some cases, the formation of LSFL-II LIPSS results in topographical properties of the surface favorable to the more hydrophilic state of the water droplet. It is difficult to distinguish what exactly causes this effect; however, it seems that the depth between the LIPSS ridges becomes lower at RL = 0.5 compared to other RL values. The depth of the LIPSS structure was 240 ± 57 nm for RL = 0.03 (SEM image insert 1 in Figure 6d), 178 ± 35 nm for RL = 0.8 (insert 2 in Figure 6d), and 100 ± 17 nm for RL = 0.5 (insert 3 in Figure 6d).

## 4. Conclusions

In this work, the wettability of a 1.4301 stainless-steel surface, structured via parallel processing using an array of 64 laser beams, was investigated. Area scanning with an 8 × 8-beam array was used to produce LIPSS in a large area during a single scanning process. The wetting control of water droplets on laser-textured stainless steel, ranging from static contact angles similar to those of the plain surface to the superhydrophobic surface with a static contact angle > 150°, was achieved. The dependence of the LIPSS period on the hatch distance (the distance between the scan lines) and irradiation fluence was determined. The relationship between the static water contact angle and the structure parameters in the Fourier plane was investigated, and a negative influence of LSFL-II LIPSS on the high surface water contact angle was found when the ratio between the LSFL-II and LSFL-I peaks in the Fourier space was close to 0.5.

## Data Availability

The data presented in this study are available from the corresponding authors upon reasonable request.
